# Severe Recurrent Pancreatitis in a Child with ADHD after Starting Treatment with Methylphenidate (Ritalin)

**DOI:** 10.1155/2014/319162

**Published:** 2014-02-11

**Authors:** Suheil Artul, Faozi Artoul, George Habib, William Nseir, Bishara Bisharat, Yousif Nijim

**Affiliations:** ^1^Department of Radiology, Nazareth Hospital, EMMS, Faculty of Medicine, Bar-Ilan University, Israel; ^2^Faculty of Medicine in the Galilee, Bar-Ilan University, Safed, Israel; ^3^Department of Nuclear Medicine, Meir Hospital, 44410 Betah Tekva, Israel; ^4^Department of Internal Medicine, EMMS Hospital, 16100 Nazareth, Israel; ^5^Pediatric Department, Nazareth Hospital, Israel

## Abstract

We present a case of a 10-year-old boy, who had severe relapsing pancreatitis, three times in two months within 3 weeks after starting treatment with methylphenidate (Ritalin) due to attention deficit hyperactivity disorder (ADHD). Pancreatitis due to the use of (methylphenidate) Ritalin was never published before. Attention must be made by the physicians regarding this possible complication, and this complication should be taken into consideration in every patient with abdominal pain who was newly treated with Ritalin.

## 1. Case Presentation

**Figure 1:**
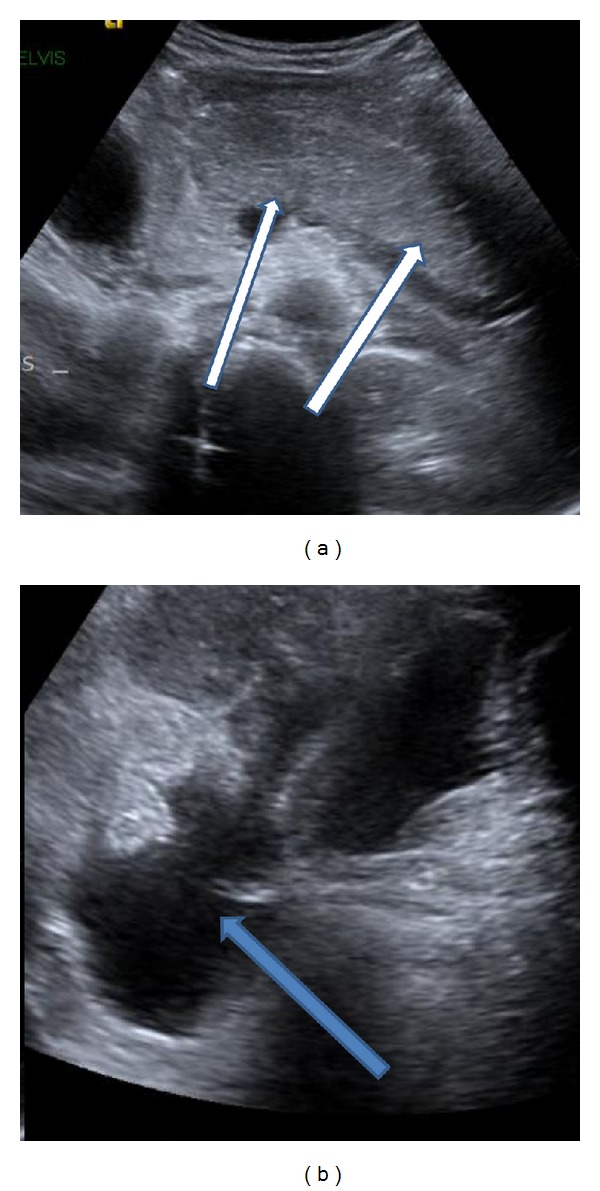
(a) Ultrasound of epigastrium region showing edematous pancreas (white arrows) and (b) ultrasound of lower abdomen showing free fluid (blue arrow).

**Figure 2:**
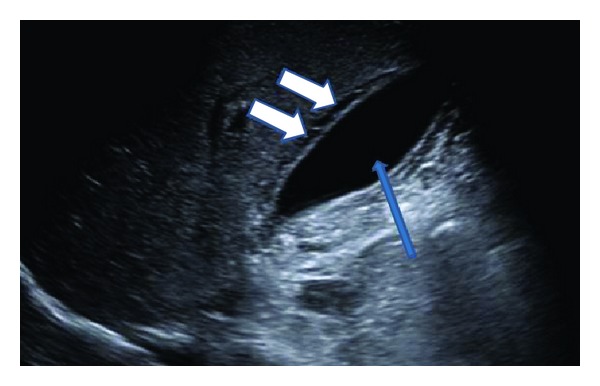
Ultrasound of the right upper quadrant showing the gallbladder free of stones (blue arrow) and thickening of gallbladder wall (white arrows).

A case of a 10-year-old boy was referred to emergency department because of an abrupt onset of aggravating abdominal pain and vomiting. The boy was generally healthy except for that he was newly diagnosed with ADHD and started the use of methylphenidate (Ritalin) for the past three weeks at a dose of 30 mg daily. Physical examination on admission revealed that the boy looks suffering and afebrile and has diffuse tenderness of abdomen without rebound and no dyspnoea. Laboratory tests showed high level of serum amylase 5824 U/L (amylase normal value: 30–110 U/L), high level of lipase 1950 U/L (normal value: 10/140 U/L), high levels of liver enzymes, AST 1259 (normal range 5–43), ALT 769 (normal range 5–40), and normal levels of electrolytes, cholesterol, triglycerides, bilirubin. There was no metabolic acidosis. Ultrasound of abdomen (Figure 1(a)) showed edematous and enlarged pancreas, big amount of free fluid in the abdomen (Figure 1(b)), thickened gallbladder wall up to 6 mm without intraluminal stones (Figure 2), and no intrahepatic or extrahepatic biliary dilatation. There was no anamnestic familial history of pancreatitis.

**Figure 3:**
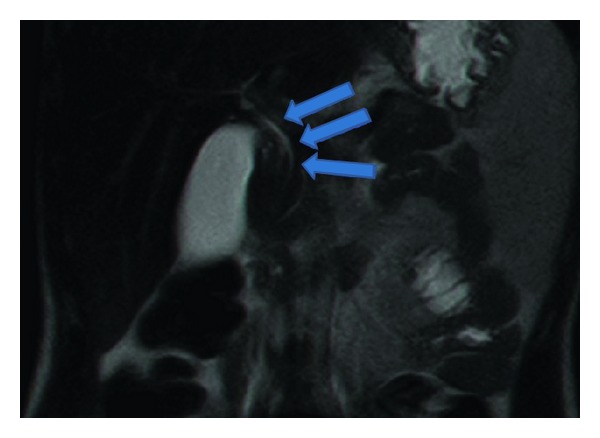
Coronal T2 MRI (as part of the MRCP STUDY) showing no dilatation and normal position of choledochus.

The boy was admitted to intensive care unit with the diagnosis of acute pancreatitis and was started workup to investigate the etiology which revealed no alcohol use, transesophageal ultrasound (EUS) followed by magnetic resonance cholangiopancreatography (MRCP) (Figure 3) no biliary stone or any congenital or acquired malformation, and normal levels of immunoglobulins which excluded autoimmune pancreatitis. Other possible causes such as viral, bacteria, and parasites screening were all negative.

The boy was treated with intravenous rehydration and fasting with nasogastric tube. The boy improved slowly and discharged with the diagnosis of idiopathic pancreatitis from hospital after one week in good condition, free of symptoms, and with normalization of laboratory tests. Three weeks later, the boy was readmitted to the hospital again with more severe similar clinical scenario, received the same palliative treatment, and discharged after two weeks with good condition. After 5 days he was readmitted again to the hospital with the same clinical presentation of severe pancreatitis. This admission lasted for one week and on discharge the family reported on the use of Ritalin and therefore it was recommended to stop taking Ritalin.

The boy is now free of symptoms for one year and half after stopping taking Ritalin.

## 2. Discussion

The use of Ritalin is noticeably increased worldwide in the last few years and is prescribed for several indications such as ADHD, behavior disorder, and even for improving scholar achievement [1].

The incidence of pediatric pancreatitis has increased significantly in the past two decades. It is estimated that 2 to 13 new cases occur annually per 100,000 children [2].

Pancreatitis affects a heterogeneous population of children, and symptoms range from mild to life threatening.

In acute pancreatitis, although the pathophysiology and functional consequences in children are identical to those observed in adults, its etiology differs significantly, although most of pediatric pancreatitis still idiopathic [3]. The common known causes of pancreatitis in children include (1) systemic diseases, such as systemic lupus erythematosus, Henoch-Schönlein purpura, Kawasaki disease, Crohn's disease, hyperlipoproteinemia, and hypertriglyceridemia; (2) different drugs and toxins, such as thiazides, furosemide, cimetidine, estrogen, and tetracycline; (3) infections; (4) obstructive diseases; (5) trauma; (6) hereditary pancreatitis; (7) autoimmune pancreatitis. In about 15% of cases the cause remains unknown after thorough investigation [4].

Unofficial data states that according to FDA reports published on the Internet in June 2013, 41 people of 8668 (0.47%) users of Ritalin in the United States declared to have pancreatitis within one month after starting the treatment. But we do not know if these 41 people have another underlying disease for developing pancreatitis.

We believe that the number of persons suffering from pancreatitis due to the use of Ritalin is more than this published case.

Physicians must pay attention regarding this possible complication and it should be taken into consideration in every patient with abdominal pain who started consuming Ritalin.

Because of clinical various degrees of presentation of pancreatitis, a lot of these patients are undiagnosed.

## 3. Conclusion

Acute pancreatitis in pediatric age could be due to the use of Ritalin. Because of increased use of this drug, physicians must be aware of this possibility and they must include this entity in the differential diagnosis in every child suffering from abdominal pain and who was also recently started to take this medicine. We suggest further investigation in this issue.

## Abbreviations

ADHD: Attention deficit hyperactivity disorder.
